# Variation in morpho-lexical development within and between diagnoses in children with neurodevelopmental disorders

**DOI:** 10.3389/fpsyg.2022.968408

**Published:** 2023-01-12

**Authors:** Susan Foster-Cohen, Toby Macrae, Jayne Newbury

**Affiliations:** ^1^Department of Linguistics, University of Canterbury, Christchurch, New Zealand; ^2^New Zealand Institute of Language, Brain and Behaviour, University of Canterbury, Christchurch, New Zealand; ^3^Christchurch Early Intervention Trust, The Champion Centre, Christchurch, New Zealand; ^4^School of Psychology, Speech and Hearing, University of Canterbury, Christchurch, New Zealand

**Keywords:** atypical language development, vocabulary, morphology, morpho-lexical, diagnosis

## Abstract

While primary diagnosis is only one aspect of the presentation of a child with neurodevelopmental delay/disorder, the degree to which early expressive language reflects diagnostic divisions must be understood in order to reduce the risk of obscuring clinically important differences and similarities across diagnoses. We present original data from the New Zealand MacArthur-Bates Communicative Development Inventory (NZCDI) from 88 English-speaking children aged 2;6 to 5;6 years receiving multidisciplinary intervention within a single family-centered program. The children had one of six pediatrician-assigned genetic or behaviorally determined diagnoses: Down syndrome (DS); motor disorders (cerebral palsy and developmental coordination disorder); global development delay; disorders of relating and communicating (R&C); other genetically defined diagnoses; or language delay due to premature (PREM) birth. Morphological and lexical development were compared within and across diagnostic groups, using both data visualization and mixed-effects modeling. Groups varied in the amount of variation within and between them, but only prematurity reached significance, in interaction with age, as a predictor of morpho-lexical scores. Further analysis of longitudinal data available from a subset of the sample (*n* = 62) suggested that individual trajectories of vocabulary growth could not be reliably predicted by diagnosis. Moreover, the distribution of word types (nouns, predicates, etc.) only distinguished PREM children with language delay from those with DS and those in the R&C group. There were strong similarities in early morpho-lexical development across these clinical populations, with some differences. These findings align with research and clinical approaches which accommodate individual variation within diagnosis, and broad similarities across diagnostic groups.

## 1. Introduction

Language development begins with words and phrases, followed by the acquisition of morphological and syntactic features (Owens, 2020). While vocabulary development is predicted by children’s cultural and communicative needs in all languages, the development of particular word types and morphology varies depending on the linguistic features of the language being acquired. In English, early development of morphology is expected to include plural and possessive “-s” on nouns, and progressive “-ing” and past tense “-ed” on verbs, as well as irregular plurals (e.g., “children”) and past tenses (e.g., “came”), with noun and verb endings often overregularized (e.g., “childs,” “comed”) as children acquire the co-occurrence patterns of language use (Brown, 1973; Bates et al., 1994). Children with a range of neurodevelopmental diagnoses access support for language delays through early intervention services. In this paper we will explore early morpho-lexical development across diagnostic populations to highlight important individual differences and between-diagnosis similarities, with a view to informing research directions and support service provision.

Diagnoses have considerable power both for clinicians and end-users and a firm early diagnosis of a neurodevelopmental disorder is usually valued by parents/caregivers of young children with language needs (Russell and Norwich, 2012; Soriano et al., 2015; Eggleston et al., 2019). However, diagnosis is also often used as a gate keeper for access to language services. In most states in the US, special education services are available only to children who have a confirmed disability that falls under a defined set of diagnostic categories, for example, specific learning disability, autism, or intellectual disability (Triano, 2000). In the UK, Dockrell and Hurry (2018) in their large scale longitudinal study of service access for elementary school-aged children with speech, language, and communication needs concluded that a purely diagnostic approach was not working well. This study will explore how children vary within diagnoses, and how they are similar across diagnoses to inform designing more equitable access routes to support services and effective individualized interventions.

Diagnoses are often assigned by a pediatrician or other medical professional prior to referral for intervention, or as part of an assessment for language delay. However, applying diagnostic categories to children can be a contentious activity. While some diagnoses associated with language delays have clear genetic markers, such as Down syndrome (DS) (Trisomy 21) (FitzPatrick and Firth, 2020), other diagnoses, such as autism or Developmental Coordination Disorder (DCD) are determined on the basis of observed behaviors against defining criteria (Fernell et al., 2013; Blank et al., 2019). Preschool children may change in their behavioral presentation over time, sometimes leading to a change in diagnosis, for example, autism to intellectual disability (Jansen et al., 2021). Some children, particularly those born prematurely, may start with language delays, but may not meet criteria for a language disorder later on (Foster-Cohen et al., 2010). For behaviorally determined disorders, there is significant debate about diagnostic criteria, boundaries and overlap with other similar disorders, including those which have clear genetic markers (Jones, 2000; Capone et al., 2006; Wilkes and Callard, 2010; Jiménez et al., 2021). It is important therefore to understand the extent to which diagnosis is a useful contribution to clinical decision making, in this case for language supports (Thomas et al., 2020; Fidler et al., 2021).

When it comes to language intervention, group studies of a single diagnostic group may not provide sufficiently nuanced understanding to guide intervention decisions or estimate children’s likely response to intervention. Studies limited to one diagnostic group ignore similarities across populations, which are apparent to clinicians who work with diverse children. For example, delayed morphology and syntax characterizes the language development of children with DS (Abbeduto et al., 2007). However, their language development is also affected by fine motor challenges to speech, similar to those with primary motor disorders (Henderson and Henderson, 2002; Wild et al., 2018; Blank et al., 2019) and/or by social and pragmatic challenges similar to those that characterize autism (Fernell et al., 2013; Lee et al., 2017) or that may be present in those born prematurely (Nguyen et al., 2018). As Perkins (2008) has argued, pragmatics is a reflection of the communicative resources available to each child. From a clinician’s perspective, the integration of multiple factors is critical.

While it is appropriate for some purposes to create phenotypical sketches of the language characteristics associated with particular diagnoses [see Hilvert and Sterling (2019) for example], the heterogeneity within a diagnosis can be so great, that grouping children by diagnosis can be less helpful than is assumed from the clinical perspective of providing language supports. Careful attention to the individual presentation within a diagnostic group is crucial if children are to be well-supported (Hippman et al., 2012; Dockrell et al., 2019). Koegel et al. (2014) and Rosenbek (2016) argued for the value of single case design research with clinical populations with a high degree of heterogeneity, as group averages, such as those reported from randomized controlled trials and meta-analyses, mask individual variation in response to intervention (for example, see Sandbank et al., 2020).

To further our understanding of clinically significant similarities and differences between and within diagnosis in early language development, the current study used a corpus of MacArthur-Bates Communicative Developmental Inventories completed by parents of children attending the same center-based pre-school early intervention program in New Zealand to explore (1) the capacity of diagnosis to predict morpho-lexical scores cross-sectionally; (2) the capacity of diagnosis to predict longitudinal growth in vocabulary size over time; and (3) the relationship between diagnostic group and vocabulary composition in terms of major word types (nouns, predicates, etc.).

## 2. Methods

The data to be presented below were gathered using the New Zealand MacArthur-Bates Communicative Development Inventory (NZCDI): words and sentences (Reese and Read, 2000; Fenson et al., 2007). This parent report measures expressive lexical and morphological development and while it is aimed at typically developing children aged 16–30 months, it is appropriate for use with older children with atypical language. The first section of the NZCDI is a checklist of 675 vocabulary items and, in the version used here, both a spoken word and sign option for each item allowing for three possible alternatives for each item (spoken word only, sign only, both word, and sign). The sign option was added to the NZCDI version used here in order to ensure that children’s full expressive vocabularies could be assessed irrespective of modality, and was particularly relevant for the children with DS in our sample who were routinely encouraged to use signed vocabulary as part of their early intervention program (Foster-Cohen et al., 2022). The second section of the NZCDI includes a list of 107 items/questions: 5 irregular plural nouns (e.g., “feet”), 20 irregular past tense verbs (e.g., “came”), 14 overregularized nouns (e.g., “feets”), 31 overregularized past tense verbs (e.g., “comed”), and a 37 item section on complexity of expression which presents pairs of utterances in which the second of the pair is more complex than the first (e.g., “Daddy car” vs. “Daddy’s car”). Parents are asked to indicate which of the pair sounds more like their child.

The NZCDI completions analyzed here were collected as part of a larger study using data collected between 2010 and 2017 exploring the perspectives of parents/caregivers of children with multi-system neurodevelopmental delays or disorders on their children’s development. With ethical approval from the Human Ethics Committee of the University of Canterbury, New Zealand, parents of children enrolled in the center were told about the study by a team member and provided with further information and informed consent documentation if they expressed an interest in being involved. The data from all those who agreed to participate has been used for this analysis. The sample to be analyzed here therefore reflects both parent interest in being involved and the program client base at the time. Parents agreed to complete the NZCDI at six monthly intervals between their entry into the study and their child graduating on to primary school. Completions were timed to coincide with a window of 2 weeks on either side of their child’s birthday and half-birthday. Because parents entered the study on a rolling basis depending on when they entered the early intervention service, some parents completed a NZCDI only once (*n* = 26), while the rest completed it two or more times (*n* = 62). Family circumstances also meant that not all parents were able to complete the measure at all the possible time points. The parents completed the paper version of the NZCDI at home in their own time and returned them to an early intervention program team member by hand or through the mail. The result was 245 completions by the parents (all mothers) of 88 children aged between 30 and 66 months (see Table 1 below).

**TABLE 1:** Participants by diagnosis, including total number of NZCDIs completed per diagnostic category, raw morpho-lexical scores and ABASII scores.

Diagnosis	Children	Total_CDI	Mean_age	Mean_vocab	SD_vocab	Mean_morpho	SD_morpho	ABAS_GAC
CP/DCD	8	17	45.18	345.24	280.40	34.47	32.51	67.35
DS	35	115	49.15	172.63	126.21	2.81	6.52	59.58
GDD	14	36	47.33	280.94	219.92	15.28	22.75	62.56
OTHER	11	27	43.33	288.48	232.28	15.93	22.13	63.73
PREM	13	34	43.68	446.50	161.33	30.44	26.39	74.88
R&C	7	16	50.63	231.31	139.97	7.75	12.57	56.44

All the children were attending the same multi-disciplinary center-based early intervention program for children with multi-system neurodevelopmental delays or disorders. They visited the center for one morning per week during school terms (up to 40 visits per year). Children always attended with their parent, and each child-parent pair received hands-on individualized therapy combined with parent coaching by each member of their therapy team at each visit. Therapy teams all included a speech and language therapist, an early intervention teacher and either a physiotherapist or occupational therapist, with additional members based on need, for example a music therapist.

All the children were referred to the program by a pediatrician who assigned a genetic or behaviorally determined multi-system primary diagnosis (where possible) prior to referral. Subsequently, all were assessed as qualifying for speech and language therapy by the program’s speech and language therapists. The numbers of children in each diagnostic category are presented in Table 1 together with the number of completions per diagnosis. Diagnoses have been grouped into six categories: (1) motor disorders: either cerebral palsy or developmental coordination disorder (CP/DCD), (2) down syndrome (DS), (3) global developmental delay (GDD) of unknown etiology, (4) other named genetic syndromes (including Williams’ syndrome and Prader-Willi syndrome) (OTHER), (5) disorders of relating and communicating (R&C) (pending a possible diagnosis of Autistic Spectrum Disorder), and (6) Children born under 1,500 gms and/or earlier than 30 weeks gestation with developmental delays (PREM), unless that delay was diagnosed as cerebral palsy, in which case they were assigned to the CP/DCD category.

As a measure of the overall developmental status of the children, parents also completed the ABASII functional development questionnaire (Harrison and Oakland, 2003) each time they completed the NZCDI. This norm-referenced questionnaire consists of 241 items covering the child’s day-to-day functioning and delivers an overall age-adjusted General Adaptive Composite (GAC) score with a mean of 100 and a standard deviation of 15. Children scoring below 85 are therefore 1 SD below the mean and those below 70 are 2 SD below the mean for their chronological age. All the groups of children in this study scored >2 SD below the mean, with the exception of the PREM group which scored >1.5 SD. The mean scores by diagnosis are included in Table 1.

To explore the children’s morpho-lexical development, the lexical and morphology sections of the NZCDI were summed separately with one point for each item checked (including one point for each second-pair checked in the complexity section) and rendered as percentages of the maximum possible on each score (675 for vocabulary and 107 for morphology). The two percentages were then averaged to give a combined morpholexical score. These were visualized by diagnostic group using overlaid plots [violin_plot() and box_plot()] in ggplot2 (version 3.3.5) in R (version 1.3.959) (Venables and Smith, 2003; Wickham, 2016).

To model the relationships in more detail, the raw lexical and morphological scores were converted to standard z-scores (ZMORPHO and ZLEX) and then combined and averaged to yield a single ZMORPHOLEX score. The set of six diagnostic options were deviation coded (DIAGNOSIS.f) and study age was z-transformed (ZSTUDYAGE). Because a simple linear model suggested that age (STUDYAGE) predicted MORPHOLEX scores (adjusted *R*^2^ = 0.07, *p* ≤ 0.001), we used a mixed effects regression model using lme4 (version 1.1-27.1) in R (Bates et al., 2015), with DIAGNOSIS.f and ZSTUDYAGE as fixed effects and a random intercepts and random slopes argument of ZSTUDYAGE|CHILD, given the uneven number of measure completions per child over time. We then used a likelihood-ratio test [anova()] to compare a model in which diagnosis and age were independent effects and one in which they interacted. This comparison suggested that the interaction model provided the best fit for the data [χ^2^(5) = 25.921, *p* ≤ 0.001]. The model to be reported is therefore: [ZMORPHOLEX ∼ DIAGNOSIS.f * ZSTUDYAGE + (1 + ZSTUDYAGE|CHILD)].

Because parents of 62 of the 88 children completed the measures two or more times, analysis of longitudinal trends in vocabulary size, growth, and composition was possible. An initial assessment of vocabulary size trajectories used a visualization of age as a predictor of percentage vocabulary size, color-coded for diagnosis. To model these relationships, a similar structure was used as above: ZLEX ∼ ZSTUDYAGE * DIAGNOSIS.f + (1|CHILD) (Again an interaction between age and diagnosis yielded a better fit than an additive model. A preliminary version of this model with a random slope argument of ZSTUDYAGE|CHILD did not converge with this data).

To explore the structure of children’s vocabularies, the individual lexical items were coded for word type (SOCIAL, NOUNS, PREDICATES, and CLOSED) using the method developed by Caselli et al. (1995). SOCIAL items include animal sounds, social routine items (such as greetings), and words for people; NOUNS includes the words for toys, body parts, food, etc.; PREDICATES are verbs and adjectives; and CLOSED are pronouns, prepositions, conjunctions, etc. Rendered as proportions of the available items in each category on the NZCDI (Pine et al., 1996; Bornstein et al., 2004), these were then plotted against overall raw vocabulary size and a bar graph of the average proportions of each word type within each diagnostic group was created. A mixed model predicting word type proportions from diagnosis, controlling for random effect of age and child was then fitted for each of the four word types: WORDPROPS ∼ DIAGNOSIS.f + (1|ZSTUDYAGE) + (1 |CHILD) (A preliminary version of this model with a random slope argument of ZSTUDYAGE|CHILD did not converge with this data). Pairwise comparisons of word type differences by diagnosis group were carried out using an estimated marginal mean package (Lenth, 2018) with Bonferroni correction.

## 3. Results

**FIGURE 1:**
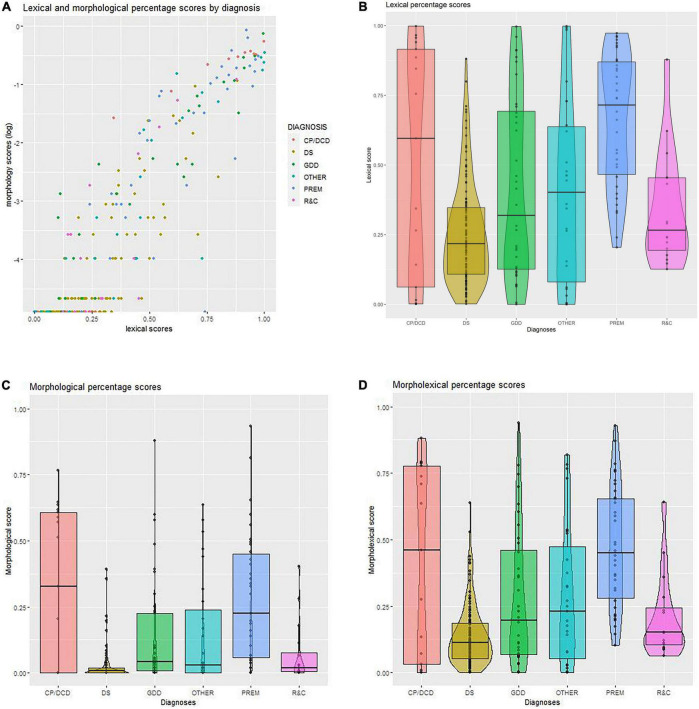
**(A)** Lexical and morphological percentage scores by diagnosis. **(B)** Lexical percentage scores. **(C)** Morphological percentage scores. **(D)** Morpholexical percentage scores. CP/DCD, cerebral palsy/developmental coordination disorder; DS, Down syndrome; GDD, global developmental delay; OTHER, other syndromes than DS; PREM, prematurely born; R&C, disorders of relating and communicating.

The means and standard deviations of raw scores on both the lexical and morphological measures are presented in Table 1. Figure 1A plots the lexical and morphological percentage scores for each child at each completion (irrespective of age). It provides a visual impression of the relationship between these scores (*R*^2^ = 0.70) as well as the distribution of scores by diagnosis, indicated by the color-coding. It suggests that children with DS tend to be clustered in the region of lower scores but that there are no obvious categorical divisions between diagnoses otherwise. The other three panels in Figure 1 present the spread of the lexical, morphological, and (combined) morpho-lexical scores for each diagnostic group, all data completions combined. While both the lexical and morphological scores reflect considerable variation, there is more variation in the lexical scores than in the morphological scores, even while the outliers in the morphological scores suggest a greater spread overall. Comparison between diagnoses is reflected in both the vertical spread of each diagnosis plot and the differences in overall shape of the violin plots. They provide evidence of both similarities and differences between diagnostic groups. In all three panels, the DS and R&C groups show the least variation, with both having the majority of scores in the lower range. The GDD group is also more clustered in the lower part of the distribution. The greatest variation is to be found within the CP/DCD group, and there is a greater number of higher scores in the PREM group.

**FIGURE 2:**
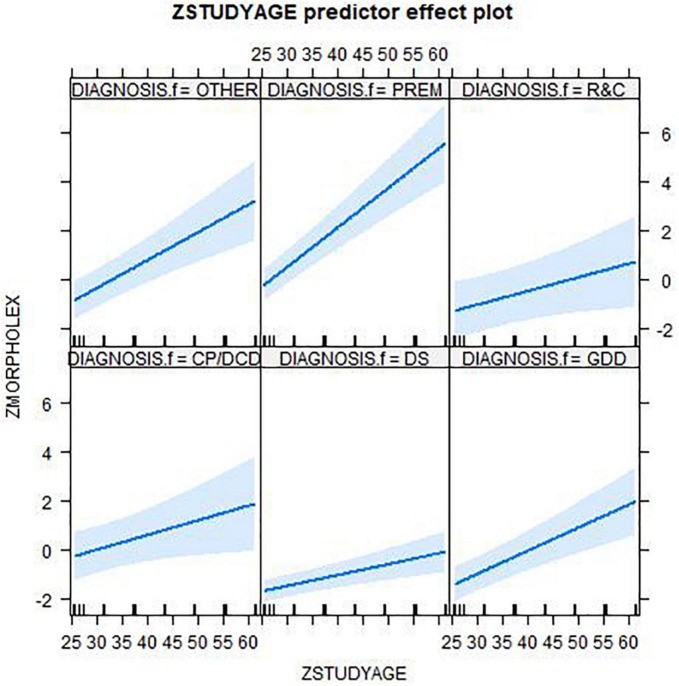
Effects plots by diagnosis from linear mixed effects models with ZMORPHOLEX dependent variable: ZMORPHOLEX ∼ DIAGNOSIS.f * ZSTUDYAGE + (1 + STUDYAGE|CHILD). Diagnoses: CP/DCD, cerebral palsy/developmental coordination disorder; DS, down syndrome; GDD, global developmental delay; OTHER, other syndromes than DS; PREM, prematurely born; R&C, disorders of relating and communicating.

Table 2 presents the output of the mixed-effects model [ZMORPHOLEX ∼ DIAGNOSIS.f * ZSTUDYAGE + (1 + ZSTUDYAGE|CHILD)]. It suggests a main effect of diagnosis reaches significance in interaction with age only in the cases of prematurity and DS. The effects plots in Figure 2, organized by diagnosis, show an upward trend relative to age for all groups. However, the slope of each group varies with the steepest slope in the PREM group and the shallowest slope in the DS group.

**TABLE 2:** Diagnoses as predictors of morpho-lexical scores (ZMORPHOLEX).

Predictors	Estimates	CI (95%)	*p*
R&C (intercept)	-3.19	−3.81 – −2.57	<0.001
CP/DCD	1.39	−0.30 – 3.07	0.107
DS	0.36	−0.49 – 1.22	0.406
GDD	-0.62	−1.81 – 0.57	0.306
OTHER	-0.54	−1.90 – 0.83	0.440
PREM	-1.09	−2.26 – 0.08	0.068
ZSTUDYAGE	0.09	0.07 – 0.11	<0.001
CP/DCD* ZSTUDYAGE	-0.03	−0.08 – 0.02	0.246
DS * ZSTUDYAGE	-0.04	−0.07 – −0.02	<0.001
GDD * ZSTUDYAGE	0.01	−0.03 – 0.04	0.732
OTHER * ZSTUDYAGE	0.03	−0.01 – 0.07	0.212
PREM * ZSTUDYAGE	0.07	0.04 – 0.11	<0.001

**FIGURE 3:**
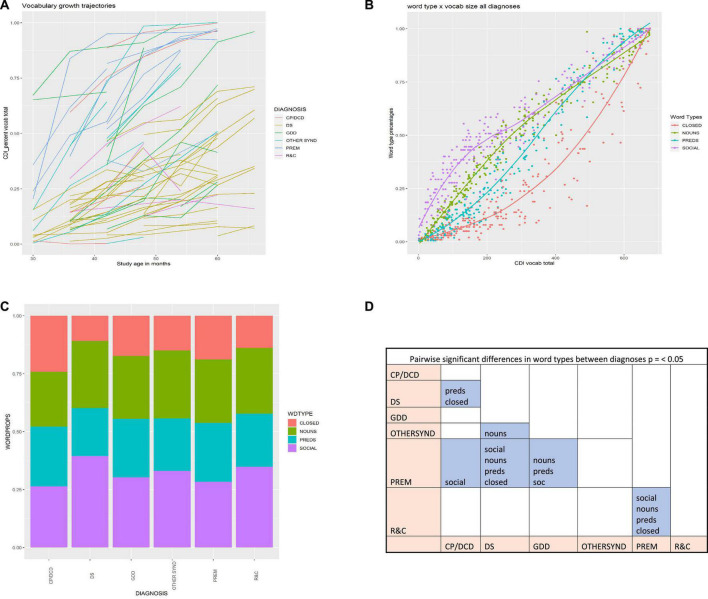
**(A)** Change over time in vocabulary size color-coded for diagnosis. **(B)** Distribution of word types by vocabulary size. SOCIAL = purple; NOUNS = green; PREDS = teal; CLOSED = red. **(C)** Distribution of word types by diagnosis. **(D)** Significant pairwise differences at *p* ≤ 0.05. CP/DCD, cerebral palsy/developmental coordination disorder; DS, Down syndrome; GDD, global developmental delay; OTHER, other syndromes than DS; PREM, prematurely born; R&C, disorders of relating and communicating.

Trajectories of overall vocabulary growth among those children with two or more data points (*n* = 62) are summarized in the Figure 3. Figure 3A shows change over time in vocabulary size color-coded for diagnosis and while it reflects slower growth over time for children with DS, it also suggests considerable variation in individual trajectories across all diagnostic groups. While some children are approaching 100% on the NZCDI by the time they are 60 or 66 months old, others remain with only a handful of items (The sparse data from children older than 60 months is due to the small number of children who transitioned to school between age 5 and 6 years. In New Zealand most children enter primary/elementary school at five, even though it is not compulsory until age six).

Figure 3B shows the distribution of word types across the span of total vocabulary sizes and suggests that the relative frequencies of the four word types (calculated as percentages of the maximum possible for each word type on the NZCDI) are more similar in the smallest and largest vocabulary sizes. Children have more of the SOCIAL words in all but the largest vocabularies. NOUNS and PREDS show similar patterns to each other with more of the NOUNS than PREDS until vocabulary size reaches around 480 items. Fewer of the CLOSED class words occur in smaller vocabularies, but reach similar relative frequencies as the other word types at the largest vocabulary sizes. Figure 3C suggests that when the same data is separated by diagnosis group, there is broad similarity across the diagnostic groups for each word type, even while there are some differences.

The analysis of pairwise differences between diagnoses for each word type frequency suggested that, at the *p* ≤ 0.001 level, the only significant difference is between children born PREM and those with DS, with the former having greater proportions of all four word types in their vocabularies than the latter, reflective of the overall differences in vocabulary size between these two groups. At the *p* ≤ 0.05 level, there are a few additional differences that nuance this picture. In particular, significant differences between those in the PREM group and those in the R&C group become visible. Figure 3D tables the pairs that have significant differences at this alpha level. Specific significant differences are that more of the SOCIAL words are present in the vocabularies of those in the PREM group than in the CP/DCD, GDD, and R&C groups. More of the NOUNS occur in the vocabularies of children in the PREM group than in the GDD and R&C groups, as well as in the vocabularies of children in the OTHER syndromes group compared to the children with DS. More of the PREDS occur in CP/DCD group than in the DS group; and in the PREM group compared to those in the GDD or R&C groups. Finally, more of the CLOSED words appear in the vocabularies of children in the CP/DCD group than in those of children with DS; and in the PREM group compared to the R&C group. Differences between all other pairs were non-significant for all word types.

## 4. Discussion

The children in this study were referred for multi-disciplinary intervention by a pediatrician in response to a range of significant and complex developmental delays and disorders. They were subsequently assessed as having language delays by speech and language therapists in the early intervention service. Children were clustered into groups on the basis of their primary diagnosis in order to explore the clinical implications of these broader divisions for language development. The aim was then to explore the capacity of primary diagnosis to predict clinically relevant similarities and differences in the morpho-lexical scores of children with a range of neurodevelopmental conditions, using a corpus of MacArthur-Bates Communicative Developmental Inventories completed by parents of children attending the same early intervention program in New Zealand. Through graphic visualization and mixed linear modeling it has provided evidence for caution in the use of diagnosis in language intervention planning for young children with neurodevelopmental conditions. The results presented here suggest morpho-lexical acquisition shows both similarities and differences within and between diagnostic categories. Children with DS and GDD tend to have the lowest scores on the NZCDI and PREMS the highest. Children with DS and R&C show the least internal variation, while those with CP/DCD show the greatest internal variation. Although age is not as strong a predictor of acquisition in this population as would be expected in a typical population, it contributes to and interacts with diagnosis to predict morpho-lexical scores for those in the DS and PREM groups. While there is a high correlation between lexical and morphological development, there is greater variation within lexical scores than morphological scores within each diagnostic category. Individual developmental trajectories for lexical development are also very varied, but children with DS show slower growth over time than other groups. Finally, an exploration of the types of words children have in their vocabularies suggests that only the PREM and DS groups are significantly different at the *p* ≤ 0.001 level with differences between the PREM and R&C groups being significant at the *p* ≤ 0.05 level. Otherwise, there are few significant differences between other pairs of diagnoses.

Our results suggest caution in using expectations from diagnosis to early morpho-lexical development as a basis for clinical judgments. While all the children in this study remained in need of language supports over time, their progress, even in the context of ongoing and frequent specialist and parent-delivered supports, could not be accurately predicted on the basis of diagnosis, even for the PREM group, who had the most positive outcomes. This study aligns with the value of research and intervention approaches which acknowledge and accommodate individual differences, such as single subject designs (Koegel et al., 2014; Rosenbek, 2016). Similarly, it supports clinically defined cohorts such as that of the STEPS data, which grouped children receiving speech pathology services by language disorder, rather than by wider diagnoses (e.g., Farquharson et al., 2015). Such approaches can help practitioners across the medical and therapeutic professions, see diagnosis as only one part of a child’s presentation; and may encourage more balanced descriptions of possible language outcomes within a condition when a diagnosis is made (Hippman et al., 2012).

While we have explored the capacity of primary, pediatrician-assigned diagnostic categories to predict morpho-lexical development in children with language delays, we have not explored latent dimensions of individual differences between children, such as non-verbal cognition, health status, gender, etc. which can be expected to impact development. Despite this, the similarities across diagnostic groups in language development were marked, and further exploration of the dimensional account for a broad range of populations of children with language delay seems to be warranted (Ellis Weismer et al., 2011; Jiménez et al., 2021). Other limitations include the uneven numbers in each diagnostic group, distributions of data points within each group, and large age-spans, despite the mixed effect model controls. While the use of parent report may be considered a further limitation, the reliability of parent report in this data has been confirmed in this and other studies (Reese and Read, 2000; Foster-Cohen and van Bysterveldt, 2016). Additionally, the fact that all the parents were attending the same early intervention program which actively encourages observation and report of children’s language gives the greatest opportunity for accurate observation of vocabulary and morphology use. Finally, morpho-lexical development is only one aspect of language development and cannot, and should not, be used in isolation in clinical assessments.

## Data availability statement

The datasets presented in this article are not readily available because at this point there is no agreement with the organization where the data were collected to share the data. This may possibly be negotiated in the future. Requests to access the datasets should be directed to corresponding author.

## Ethics statement

The studies involving human participants were reviewed and approved by Human Ethics Committee, University of Canterbury. Written informed consent to participate in this study was provided by the participants’ legal guardian/next of kin.

## Author contributions

SF-C collected the data and conducted the analysis with input from TM and JN. All authors contributed equally to the interpretation of the results and the writing of the manuscript, contributed to the article, and approved the submitted version.
